# Attitudes of inner city patients with cardiovascular disease towards meditation

**DOI:** 10.15761/JIC.1000152

**Published:** 2016-03-10

**Authors:** Amit J. Shah, Robert J. Ostfeld

**Affiliations:** 1 Assistant Profesor, Department of Epidemiology, Rollins School of Public Health, Emory University, Atlanta GA, USA; 2 Assistant Professor, Department of Medicine, Division of Cardiology, Emory University School of Medicine, Atlanta, GA, USA; 3 Staff Physician, Division of Cardiology, Altanta Veterans Affairs Medical Center, Atlanta, GA, USA; 4 Associate Professor of Clinical Medicine, Department of Medicine, Division of Cardiology, Montefiore Medical Center, Albert Einstein College of Medicine, Bronx NY, USA

**Keywords:** Prevention, Preventive Cardiology, Meditation, Attitudes, Heart Disease

## Abstract

**Objectives:**

Meditation is a stress reduction intervention that is of potential benefit to patients with cardiovascular disease, but its interest in inner city cardiology patients is unknown. We surveyed patients at an inner city cardiology clinic about their interest in learning meditation and the impact of stress on their lifestyles.

**Methods:**

A survey was distributed to 215 consecutive patients in an inner city cardiology clinic. Chi-square tests were used to compare those interested and not interested in meditation.

**Results:**

Of the 215 surveys, 54 were excluded because of 2 or more missing responses, leaving 161 for analysis. The mean age was 61 (+/− 16.5) years; 59% were female, 37% were black non-Hispanic, and 34% were Hispanic, and 18% were white. Overall, 46% expressed interest in learning meditation, and 64% agreed that less stress would facilitate living a healthy lifestyle. In subgroup analysis, the highest levels of interest in meditation classes occurred in patients who were younger than 65 years old (69% interested) and those who agreed that less stress would facilitate living a healthy lifestyle (71% interested).

**Conclusion:**

Many of the patients at an inner city cardiology clinic may have interest in learning meditation. Given the effects of stress in this population, clinical trials involving meditation are warranted.

## Introduction

Chronic mental stress is common amongst patients with cardiovascular disease, and may be a significant contributor to its development [1,2]. Those with depression after the development of coronary artery disease have higher mortality than those without depression [3]. Anti-depressants and cognitive behavioral therapy, however, have shown limited efficacy in reduction of mortality after myocardial infarction [4]. Meditation, which can induce a state of relaxation and equanimity, is a complementary technique that may be efficacious for mental stress, depression [5] and secondary prevention in patients with CAD [6].

Although meditation techniques such as mindfulness based stress reduction (MBSR) and transcendental meditation (TM) have shown efficacy in improving various health measures [5,7], it is a learned technique that requires significant time and dedication on the part of the learner. The population-wide effectiveness of meditation depends critically on the interest and motivation of the patient population [8]. Studies have shown varied degrees of interest of people in the community [9] as well as those with lower back pain [10], but have not examined patients with cardiovascular disease. In this study, we perform a descriptive analysis on outpatients in an inner city cardiology clinic that explores their attitudes and beliefs regarding stress and meditation. These findings may be particularly useful for those planning and recruiting for clinical trials, as well as for those potentially offering clinical services for meditation in inner city cardiology clinics, where relatively few patients may have resources to seek such treatment and pay out of pocket.

## Methods

In the Montefiore Medical Center outpatient cardiology clinic in the Bronx, NY, 215 consecutive English speaking patients were asked by the receptionist to fill out an anonymous questionnaire prior to their visit from 2007–2008. Questions answers were written in Likert scale format. Interest in meditation was ascertained by asking patients if they would attend a free meditation class or listen to a meditation audio tape or compact disc (CD). Classes were described as “free” to assess for interest independent of financial resources. Interest in meditation through portable means (audio CD) was also assessed as an alternative to classes for those with limited mobility. Patients were asked about their opinions of stress, and its relationship to unhealthy behaviors, as this may relate to their attitude towards meditation. They were also asked about their knowledge of meditation, given that insufficient knowledge about meditation may be a strong reason for disinterest. The survey also assessed self-rated health status using a validated question from the Center for Disease Control Health Related Quality of Life Survey [11] to ascertain if poor health may or may not provide a motivational role in meditation readiness. Surveys with more than two unanswered questions were discarded.

The data was analyzed using SPSS 16.0 software. Subgroup analysis was done using chi-square tests for binary variables to compare the interest in meditation for various categories. Non-responses were excluded. Those who answered “don’t know” were also excluded from subgroup analysis because of possible misclassification bias.

## Results

Of the 215 patients who were asked to complete the survey, 170 completed it, and 9 were discarded because of insufficient responses. In total, 161 were included in this analysis. The mean age was 61 +/− 16.5 years, and most respondents were Black (37%) or Hispanic (34%); 49% rated their health as fair or poor. Table 1 summarizes the responses to the Likert scale questions; 47% agreed that stress affects their behaviors, and 64% agreed that less stress would make it easier for them to live a healthier lifestyle. Additionally, 73% agreed or strongly agreed that they knew the meaning of meditation, 46% expressed interest in a CD or audio tape that teaches them ways to relax, and 45% expressed interest in going to a free meditation class.

Those who answered “agree” or “strongly agree” to interest in meditation classes or portable audio were compared to those who answered “neutral,” “disagree,” or “strongly disagree” for various subgroups (Table 2). Subgroups which expressed a significantly higher level of interest in meditation classes included patients who: were younger (vs. older) than 65 years old, associated stress with unhealthy behaviors, and felt they understood the meaning of the word “meditation.” With regards to portable audio that teaches relaxation, subgroups with significantly higher interest levels included patients who: were younger (vs. older) than 65 years old, black non-Hispanic and Hispanic (vs. white non-Hispanic), and felt that less stress would allow them to live a healthier lifestyle.

## Discussion

In this cohort of inner city cardiology clinic patients, nearly half expressed an interest in learning about meditation. Patients who expressed the highest interest in meditation classes and portable audio that teaches relaxation included those who were younger than 65 years old, as well as those who agreed that stress reduction facilitates living a healthy lifestyle. To the best of our knowledge, this is the first study to query inner city cardiology outpatients about their attitude towards meditation and stress, and an important first step towards evaluating the effectiveness of mediation in this high risk population.

Stress appears to play a role in the unhealthy behaviors of these patients, given that 64% of patients answered “agree” or “strongly agree” to the statement “if I felt less stress, living a healthy lifestyle would be easier.” Furthermore, given that 72% of patients who agreed with that statement also reported interest in meditation classes (excluding those who answered “don’t know), it is possible that patients who believe stress is a burden on their lifestyle may be particularly motivated to learn meditation. The finding that younger patients are more highly interested in meditation than older patients is consistent findings from a national survey looking at practice patterns in the United States [12] and likely reflect a difference in attitudes, understandings, or beliefs in the younger compared to the older population.

Few similar studies have been done in other populations. In a random sample of 416 middle class, non-minority residents from Ontario, Canada, 33% reported they would use holistic therapy (Lewis et al., 2001). This is lower than the level of interest found in our study. On the other hand, a study of patients with lower back pain found that only 4% of patients reported that they would be willing to participate in a trial involving meditation for lower back pain, and only 27% would try it if their provider thought it was reasonable (Sherman et al., 2004). These findings show that the degree of interest in meditation depends on both the population studied and indication for trying it. Patients with cardiovascular disease may, in general, be more interested in learning meditation than other patient populations, although this is yet to be studied.

Although nearly 50% of the population we studied showed interest in meditation, only 8% of people in the United States with cardiovascular disease have practiced meditation within the last 365 days [13]. It is possible that this number may increase if more opportunities to learn meditation are offered to patients. Physicians may also play a motivational role by educating patients about the possible cardiovascular benefits of stress reduction, and the evidence regarding stress reduction through meditation. However, it is unclear if these actions will increase the proportion of those who meditate or decrease cardiovascular outcomes; further study is needed.

The survey study has several limitations. Respondents were limited to those with sufficient literacy in English. A substantial proportion (up to 22% for one question) of the patients answered “Don’t Know,” which further limits the survey’s generalizability and positively biased the results presented in Table 2, in which such people were excluded. Results may also be influenced by a social desirability response bias, in which patients are biased towards agreeable responses. Finally, in describing the meditation class as “free,” our results do not apply to fee-based meditation classes and portable audio.

## Conclusion

A moderate interest in learning meditation exists in this inner city cardiology population, and attitudes toward stress and its effects on behavior, as well as understanding of meditation, are motivational factors. Studies investigating whether this interest in meditation can be translated into patients adopting these techniques for general stress reduction, and whether this change improves health outcomes, are warranted.

## Figures and Tables

**Table 1:** Summary of responses to questions 1–5 with n (%) and median with interquartile range.

Question	Strongly Disagree (1)	Disagree (2)	Neutral (3)	Agree (4)	Strongly Agree	Don’t Know (0)	Median (IQR)
Stress can cause me to do unhealthy things, such as smoke cigarettes and eat unhealthy foods	24 (15%)	30 (19%)	18 (11%)	49 (30%)	28 (17%)	11 (7%)	**4 (2,4)**
If I felt less stress, living a healthy lifestyle would be easier (for example, dieting and exercise)	8 (5%)	9 (6%)	25 (15%)	69 (43%)	34 (21%)	14 (9%)	**4 (3,4)**
I understand the meaning of the word “meditation”	4 (3%)	6 (4%)	9 (9%)	82 (51%)	35 (22%)	19 (12%)	**4 (4, 4.25)**
I would have interest in a CD or audio tape that teaches me ways to relax	8 (5%)	12 (12%)	30 (19%)	40 (25%)	33 (21%)	12 (13%)	**4 (3, 5)**
If a free meditation class were offered near my house, I would go to it	7 (4%)	23 (14%)	22 (14%)	46 (29%)	26 (16%)	35 (22%)	**4 (3, 4)**

Abbreviations: IQR=interquartile range

**Table 2:** Comparison of the interest of various subgroups in meditation classes and portable audio (compact disc).

	Meditation Classes (n=116)			Relaxation Portable Audio (n=131)		
	Interested	Not Interested	p	Interested	Not Interested	p
Age						
Less than 65 years old	69.0%	31.0%	0.01	63.9%	36.1%	0.05
Greater than or equal to 65 years old	42.3%	57.7%		45.8%	54.2%	
Gender						
Male	61.5%	38.5%	0.58	55.8%	44.2%	0.86
Female	55.9%	44.1%		57.9%	42.1%	
Race/Ethnicity						
Black Non-Hispanic	66.0%	34.0%	0.11	65.4%	34.6%	0.01
White Non-Hispanic	42.9%	57.1%		29.2%	70.8%	
Other Non-Hispanic	76.9%	23.1%		42.9%	57.1%	
Hispanic	51.2%	48.8%		63.4%	36.6%	
General Health						
Poor/Fair/Good	55.9%	44.1%	0.43	53.6%	46.4%	0.493
Very Good/Excellent	59.3%	40.7%		55.4%	44.6%	
Stress can cause me to do unhealthy things, such as smoke cigarettes and eat unhealthy foods						
Agree or Strongly Agree	72.1%	27.9%	0.01	65.2%	34.8%	0.103
Neutral, Disagree, or Strongly Disagree	49.1%	50.9%		50.0%	50.0%	
If I felt less stress, living a healthy lifestyle would be easier (for example, dieting and exercise)						
Agree or Strongly Agree	71.3%	28.7%	<0.001	66.7%	33.3%	0.001
Neutral, Disagree, or Strongly Disagree	27.3%	72.7%		31.3%	68.7%	
I understand the meaning of the word “meditation”						
Agree or Strongly Agree	65.3%	34.7%	0.05	60.0%	40.0%	0.12
Neutral, Disagree, or Strongly Disagree	40.9%	59.1%		38.9%	61.1%	

## Abbreviations

MI: myocardial infarction
MBSR: mindfulness based stress reduction
TM: transcendental meditation
